# Prevalence of Abdominal Obesity in Spanish Children and Adolescents. Do We Need Waist Circumference Measurements in Pediatric Practice?

**DOI:** 10.1371/journal.pone.0087549

**Published:** 2014-01-27

**Authors:** Helmut Schröder, Lourdes Ribas, Corinna Koebnick, Anna Funtikova, Santiago F. Gomez, Montserat Fíto, Carmen Perez-Rodrigo, Lluis Serra-Majem

**Affiliations:** 1 Cardiovascular Risk and Nutrition Research Group (CARIN), REGICOR Study Group, Research Programme in Inflammatory and Cardiovascular Disorders (RICAD), IMIM (Hospital del Mar Medical Research Institute), Barcelona, Spain; 2 CIBER Epidemiology and Public Health (CIBERESP), Instituto de Salud Carlos III, Madrid, Spain; 3 Fundación para la Investigación Nutricional (Nutrition Research Foundation), Barcelona, Spain; 4 CIBER Physiopathology of Obesity and Nutrition (CIBEROBN), Instituto de Salud Carlos III, Madrid, Spain; 5 Department of Research and Evaluation, Kaiser Permanente Southern California, Pasadena, California, United States of America; 6 Fundación THAO, Barcelona, Spain; 7 Community Nutrition Unit, Bilbao Department of Public Health, Bilbao, Spain; 8 Department of Clinical Sciences, University of Las Palmas de Gran Canaria, Las Palmas de Gran Canarias, Spain; University of Barcelona, Faculty of Biology, Spain

## Abstract

**Background:**

Evidence indicates that central adiposity has increased to a higher degree than general adiposity in children and adolescents in recent decades. However, waist circumference is not a routine measurement in clinical practice.

**Objective:**

This study aimed to determine the prevalence of abdominal obesity based on waist circumferences (WC) and waist to height ratio (WHtR) in Spanish children and adolescents aged 6 to 17 years. Further, the prevalence of abdominal obesity (AO) among normal and overweight individuals was analyzed.

**Design:**

Data were obtained from a study conducted from 1998 to 2000 in a representative national sample of 1521 children and adolescents aged 6 to 17 years (50.0% female) in Spain. WC and WHtR measurements were obtained in addition to BMI. AO was defined as WHtR ≥0.50 (WHtR-AO), sex and age specific WC≥90^th^ percentile (WC-AO1), and sex and age specific WC cut-off values associated with high trunk fat measured by by dual-energy X-ray absorptiometry (WC-AO2).

**Results:**

IOTF- based overweight and obsity prevalence was 21.5% and 6.6% in children and 17.4% and 5.2% in adolescents, respectively. Abdominal obesity (AO) was defined as WHtR≥0.50 (WHtR-AO), sex- and age-specific WC≥90th percentile (WC-AO1), and sex- and age-specific WC cut-off values associated with high trunk fat measured by dual-energy X-ray absorptiometry (WC-AO2). The respective prevalence of WHtR-AO, WC-AO1, and WC-AO2 was 21.3% (24.6% boys; 17.9% girls), 9.4% (9.1% boys; 9.7% girls), and 26.8% (30.6% boys;22.9% girls) in children and 14.3% (20.0% boys; 8.7% girls), 9.6% (9.8% boys; 9.5% girls), and 21.1% (28.8% boys; 13.7% girls) in adolescents.

**Conclusion:**

The prevalence of AO in Spanish children and adolescents is of concern. The high proportion of AO observed in young patients who are normal weight or overweight indicates a need to include waist circumference measurements in routine clinical practice.

## Introduction

The childhood obesity epidemic is one of the greatest current challenges for health policy. Two surrogate measures of abdominal adiposity, waist circumference (WC) and waist-to-height ratio (WtHR), have been related to cardiometabolic risk in children and adolescents [1] and threshold measurements have been proposed to identify at-risk children [2]. In the latter study, the prevalence of abdominal obesity varied from 3.8% to 33.2% in the countries studied. This wide range is due in part to actual population differences and in part to differences in surrogate measures of abdominal adiposity and the cut-off points used to define abdominal obesity. Furthermore, the availability of nationally representative data on the prevalence of abdominal obesity in children is limited, obscuring the potential magnitude of the problem.

Elevated abdominal fat within the weight ranges defined as normal and overweight is a strong risk factor for cardiovascular disease and premature mortality adults [3], [4]. Data from the Bogolusa Heart Study showed a high cardiometabolic risk among normal and overweight children with abdominal obesity compared to overweight children without excessive abdominal fat accumulation [5]. However, fat distribution is not routinely measured in clinical practice. Instead, national and international guidelines recommend the use of percentile cut-off points of weight and height based on country-specific growth charts to identify children and adolescents at cardiometabolic risk [6], [7]. Secular trends of body mass index (BMI) and WC indicate greater increase in abdominal fat compared to general adiposity [8]–[11]. Furthermore, a representative study from the Balearic Islands showed that 20% of abdominally obese adolescents were classified as overweight [12]. Therefore, we hypothesized that a nationwide analysis would show an important proportion of normal and overweight children with elevated levels of abdominal adiposity.

This study had two objectives: 1) To determine the prevalence of abdominal obesity based on WC and WHtR measurements in a representative national sample of Spanish children and adolescents aged 6 to 17 years and 2) To analyze the proportion of abdominal obesity in normal and overweight young Spaniards. Based on current screening procedures and recommendations, these children and adolescents are unlikely to be characterized as being at high cardiometabolic risk.

## Methods

### Study design and subjects

Parental written consent on behalf of each participant under 18 years was obtained. The study protocol and was approved by the ethics committee of the Spanish Society of Community Nutrition. The enKid study on nutritional status and food habits of Spanish children and young people, conducted between 1998 and 2000, was a cross-sectional survey of the Spanish population aged 2 to 24 years (n = 3534), selected by multistage random sampling procedures based on a population census. The theoretical sample size was set at 5500 individuals, taking into account an anticipated 70% participation rate, which would result in a sample of approximately 3.850 individuals. The objective of the EnKid study was two-fold: 1) to establish the prevalence of micronutrient deficiencies among the population aged 2 to 24 years and 2) to analyze the association of these micronutrients with group membership. The sample size was calculated according to 1) the estimated prevalence of most micronutrients with 95% confidence interval and accuracy of +/− 5% of the average value of the micronutrient and 2) a statistical power of 80% to detect significant differences between two groups > = 10% of the mean of the micronutrients (setting the alpha error at p< = 0.05). The sample size was overestimated by 30% to cover possible losses due to census errors.

The present study included 1521 participants aged 6 to 17 years.

### Data collection

Home interviews were conducted by 43 dietitians and nutritionists who had undergone a rigorous selection and training process to standardize the data collection. Survey data were entered by the same field staff into laptop computers with software specifically designed for the study. Completed interview data were periodically sent to the co-ordinating centers in Barcelona and Bilbao.

### Antropometric measurements

The anthropometric variables were gathered in the domicile of the participant. All the interviewers received the same theoretical/practical standardized formation for the anthropometric measurements in a workshop. During this workshop all interviewers collected weight, height, and waist circumferences in the same model to evaluate the uniformity of data collection.

Body weight, height, and waist circumferences were measured on the day of the interview, with the subject in underwear without shoes, using an electronic scale (to the nearest 100 g), a portable Kawe stadiometer (to the nearest 1 mm), and a Hoechst metric tape (to the nearest 1 mm), respectively. Measuring devices were systematically calibrated.

### Reference curves

Waist circumference reference curves were calculated. To obtain the WC percentiles by age, a quantile regression spline was performed separately in boys and girls, using the rqss from quantreg [13], R package [14]. The technical details used to fit these models are described by Koenker et al. [15]. The degree of smoothness measured by λ was chosen by minimizing the Akaike Information Criterion (AIC) of the model, optimizing the balance between smoothness and data fitting as suggested in [16]. The sex- and age-specific 90th percentile of WC was calculated.

### Definition of obesity and abdominal obesity

Excessive abdominal fat was defined as a) WHtR >0.50 [17] (WHtR-AO) b) equal to or greater than the sex- and age-specific 90^th^ percentile of WC (WC-AO1), and c) sex and age specific WC cut-off values associated with high trunk fat measured by by dual-energy X-ray absorptiometry (WC-AO2) [18].

The BMI categories followed Cole et al. [19]. Spanish recommendations for identifying overweight and obese children and adolescents are based on growth curves from [20]. We presented data on BMI categories based on the IOTF criteria [19] to facilitate international comparision of our data. In Spain, tables published by the F. Orbegozo Foundation [20] are frequently used in routine clinical practice to define BMI categories. Therefore, we additionally included data based on this definition. Both categories were used in our analysis. We presented data on BMI categories based on the IOTF criteria to facilitate international comparision of our data. In Spain, tables published by the Fundacion F. Orbegozo are frequently used in routine clinical practice to define BMI categories. Therefore, we additionally included data based on this definition.

### Statistical analysis

Comparison of proportions of abdominal obesity and sociodemographic variables was performed by χ^2^ test. Differences were considered significant if *P*<0•05. Statistical analysis was performed using SPSS version 18•0. (SPSS Inc. Chicago, Ill., USA). The software program R was used to fit waist reference curves.

## Results

Prevalence of WHtR-abdominal obesity was higher than general obesity based on IOTF definition. Mean WC and WHtR for boys and girls aged 6 to 11 years were 64.1±8.3 cm, 0.473±0.05 cm/cm, 61.6±7.1 cm, and 0.455±0.05 cm/cm, respectively. In adolescent boys and girls aged 12 to 17 years we found a mean WC and WHtR of 76.7±10.4 cm, 0.457±0.06 cm/cm, 69.5±7.7 cm, and 0.429±0.05 cm/cm, respectively. A higher proportion of children had WHtR-AO and WC-AO2 compared to adolescents (Table 1). Among adolescents, more boys than girls had WHtR-AO and WC-AO2. In this age group, WC-AO1 was more prominent in the south and Canary Islands than in central and north Spain (Table 1) but was less prevalent nationwide than WHtR-AO in the normal and overweight categories (Table 1). The prevalence of abdominal obesity among normal weight, overweight, and obese children and adolescents strongly depends on the definition of abdominal obesity and BMI categories (Table 2).

**Table 1:** Prevalence of abdominal obesity in children and adolescents from Spain^1^.

	6 to 11 years (n = 587)			12 to 17 years (n = 933)		
	WC^2^	WC^3^	WHtR^4^	WC^2^	WC^3^	WHtR^4^
All	13.0 (10.3;15.8)	26.8 (23,2;30.4)^5^	21.3 (18.1;24.8)^5^	11.6 (9.5;13.7)	21.1 (18.5;23.8)	14.3 (12.1;16.6)
Boys	14.3 (10.3;18.3)	30.6 (25.5;35.7)^6^	24.6 (19.9;29.8)	12.2 (9.3;15.3)	28.8 (25.1;32.5)^6^	20.0 (16.5;23.0)^6^
Girls	11.8 (8.1;15.5)	22.9 (17.8;28.0)^5^	17.9 (13.6;22.4)^5^	10.9 (8.1;13.7)	13.7 (10.;17.3)	8.7 (6.2;11.3)
Community size						
<10000	12.4 (6.7;18.1)	29.5 (21.8;37.1)	20.9 (13.8;28.1)	11.7 (7.4;15.9)	20.2 (14.8;25.5)	11.2 (6.6;15.8)
10000–49999	13.5 (7.8;19.1)	24.5 (17.6;31.4)	22.0 (15.2;28.8)	12.6 (8.6;16.7)	17.6 (12.4;22.7)	18.4 (14.1;22.6)
50000–350000	10.6 (5.9;15.4)	26.2 (18.9;33.6)	19.5 (13.1;25.9)	9.0 (5.4;12.6)	22.6 (17.6;2.6)	11.5 (7.1;15.9)
>350000	15.7 (9.9;21.4)	27.5 (20.4;34.5)	23.5 (17.0;30.1)	13.4 (8.6 (18.2)	24.7 (19.0;30.5)	16.4 (11.5;21.3)
Region						
Center	18.7 (11.3;26.1)	34.9 (26.5;43.3)	30.8 (23.1;38.6)	11.7 (7.4;16.0)	20.1 (14.6;25.6)	16.4 (11.7:21.1)
Northeast	10.3 (5.3;15.2)	17.8 (10.7;24.9)	20.0 (13.3;26.7)	6.4 (3.1;9.8)	15.8 (10.2;21.5)	11.3 (6.5;16.2)
North	9.5 (4.7;14.2)	22.3 (15.;29.4)	18.2 (11.6;24.9)	10.3 (6.4;14.3)	20.3 (15.0;25.5)	10.8 (6.3;15.3)
South	13.3 (6.5;20.0)	29.6 (20.9;28.3)	19.4 (11.2;27.5)	16.5 (10.4;22.7)	29.5 (22.7;36.3)	18.8 (13.0;24.7)
Levante	15.2 (6.5;23.8)	37.9 (27.3;48.5)	19.7 (9.8;29.6)	13.9 (7.4;20.4)	22.2 (14.5;29.9)	16.7 (10.0;23.3)
Canary Islands	22.2 (3.0;41.4)	33.3 (13.0;53.7)	22.2 (3.2;41.2)	25.0 (9.0;41.0)	28.6 (13.5;43.7)	20.7 (7.9;33.5)

1 Values are presented in percentage (95% confidence interval) ^2^Waist circumferences above the sex- and age-specific 90^th^ percentile.^3^Waist circumference cut-off according yo Taylor et al. [18] ^4^ Waist to height ratio ≥0.50^5^ p<0.05 between age groups^6^ p<0.05 within age group.

**Table 2:** Prevalence of abdominal obesity according to BMI categories.

	IOTF^1^			BMI categories Spain^2^		
	Normal weight	Overweight	Obesity	Normal weight	Overweight	Obesity
**6–11 years**						
WC^3^						
all	2.2 (0.8;3.6)	31.7 (23.6;39.9)	66.7 (51.9;81.5)	2.9 (1.3;4.5)	15.9 (6.8;24.9)	52.5 (42.7;62.2)
boys	0	36.2 (24.9;47.6)	76.2 (58.0;94.4)	0	13.3 (3.4;23.3)	60.3 (47.8;72.9)
girls	3.8 (1.2;6.3)	26.3 (14.9;37.7)	55.6 (32.6;78.5)	4.8 (2.1;7.6)	22.2 (3.0;41.4)	41.9 (27.1;56.6)
WC^4^						
all	9.6 (6.8;12.5)	67.5 (59.2;75.8)	82.1 (70.1;94.1)	10.1 (7.2;13.0)	49.2 (36.9;61.6)	81.2 (73.6;88.8)
boys	10.3 (6.2;14.5)	71.0 (60.3;81.7)	95.2 (86.1;104.3)	9.5 (5.3;13.6)	46.7 (32.1;61.2)	87.9 (79.5;96.3)
girls	9.0 (5.1;12.8)	59.6 (46.9;72.4)	66.7 (44.9;88.4)	10.6 (6.6;14.6)	55.6 (32.6;78.5)	72.1 (58.7;85.5)
WHtR^5^						
all	7.5 (4.9;10.0)	49.2 (40.5;57.9)	82.1 (70.0;94.1)	8.4 (5.7;11.1)	39.7 (27.6;51.8)	64.4 (55.0;73.7)
boys	6.4 (3.0;9.8)	59.4 (47.8;71.0)	90.5 (77.9;103.0)	5.3 (2.1;8.4)	37.8 (23.6;51.9)	79.3 (68.9;89.7)
girls	8.5 (4.7;12.2)	36.8 (24.3;49.4)	72.2 (51.5;92.9)	11.0 (6.9;15.1)	80.0 (55.2;104.8)	79.3 (68.9;89.7)
**12–17years**						
WC^3^						
all	1.7 (0.7;2.6)	31.3 (24.1;38.4)	89.6 (80.9;98.2)	1.3 (0.5;2.1)	16.9 (9.1;24.6)	60.4 (52.7;68.7)
boys	0	22.3 (14.3;30.4)	86.5 (75.5;97.5)	0	11.5 (2.9;20.2)	55.7 (45.3;66.1)
girls	2.8 (1.2;4.4)	47.4 (34.4;60.3)	100	2.1 (0.7;3.5)	24.3 (10.5;38.1)	69.6 (56.3;82.9)
WC^4^						
all	5.3 (3.7;7.0)	67.5 (60.2;74.8)	97.9 (93.9;102.0)	5.3 (3.6;7.0)	43.8 (33.5;54.1)	87.3 (81.7;92.9)
boys	6.1 (3.4;8.7)	73.8 (65.3;82.3)	97.3 (92.1;102.5)	7.0 (4.2;9.8)	53.8 (40.3;67.4)	92.0 (86.4;97.7)
girls	4.8 (2.7;6.9)	56.1 (43.3;69.0)	100	3.9 (2.0;5.9)	29.7 (15.0;44.5)	78.3 (66.3;90.2)
WHtR^5^						
all	1.8 (0.8;2.8)	44.1 (36.4;51.8)	97.9 (93.9; 102.0)	1.6 (0.7;2.5)	19.1 (10.9;27.3)	76.3 (69.1;83.5)
boys	3.2 (1.2;5.1)	44.7 (31.1;54.3)	97.3 (92.1;102.5)	3.2 (1.2;5.1)	21.2 (10.1;32.3)	80.7 (72.4;88.9)
girls	0.8 (−0.1;1.6)	43.1 (30.4;55.8)	100	0.3 (−0.0;0.8)	16.2 (4.3;28.1)	68.1 (54.8;81.4)

1 Prevalence calculated using IOTF reference values (cut-off points corresponding to an adult BMI≥25 and <30 kg/m2). Normal weight (70.9%; 76.6%), overweight (21.5%;17.4%), and obesity (6.6%;5.2%) (children; adolescents) [19].

2 Normal weight (71.0%; 75.0%), overweight (10.7%;9.7%), and obesity (17.2%;14.5%) (children; adolescents) according to Hernandez [20].

3 Waist circumferences above the sex and age specific 90^th^ percentile.

4 Sex and age specific WC cut-off values associated with high trunk fat measured by by dual-energy X-ray absorptiometry [18].

5 Waist to height ratio ≥0.5.

## Discussion

Abdominal obesity, based on a WHtR ratio of equal to or greater than 0.50, and on WC cut-off values associated with high trunk fat measured by dual-energy X-ray absorptiometry, was highly prevalent in Spanish children and adolescents. A considerable proportion of normal and overweight children were abdominally obese and, thus, at risk of obesity associated comorbidities.

Prevalence rates of abdominal obesity based on both WC and WHtR measures exceeded that of general obesity based on IOTF classification in Spanish children and adolescents. However, there is no consensus about an international WC threshold for abdominal obesity in children and adolescents. This likely contributed to the wide range of abdominal obesity that has been reported for national populations. Few national representative surveys have reported obesity prevalence in adolescents aged 10 to 18 years, but reports ranged from 3.8% 33.2% [2]. Compared to most of those surveys, abdominal obesity based on the 90^th^ percentile cut-off, but not the cut-off proposed by Taylor et al [18], was less prevalent in the present population.

De Moraes and colleagues [2] indicate that most studies they reviewed did not use the appropriate statistical method to determine WC cut-offs. In the present study, the prevalence of abdominal obesity was considerably increased because the cut-off thresholds were based on the association between WC and high trunk fat measured by dual-energy X-ray absorptiometry. In the absence of internationally validated cut-offs for abdominal obesity, however, it is difficult to interpret this finding. Furthermore, estimation of the true prevalence of abdominal obesity is affected by differential and non-differential misclassification. The consequence is an unpredictable over- or underestimation of the true prevalence of abdominal obesity in a population

It has been shown that the WHtR correctly discriminates between low and high levels of total and central fat measured by dual-energy X-ray absorptiometry in children and adolescents [21], [22]. Furthermore, abdominal adiposity defined by the WHtR is related to cardiometabolic risk in children and adolsecents [23]–[26]. Two recently published studies demonstrate the predictive value of WHtR for detecting cardiometabolic risk [25], [26]. However, neither of these studies found WHtR to have a better predictive capacity than WC and BMI. The WHtR has gained increasing interest as a simple measure of abdominal adiposity, independent of sex and age [17]. A cut-off of 0.50 has been proposed, based on studies of the association between WHtR and cardiometabolic risk in adults [17], [27]. The weak association of this ratio with age seems to partially justify applying the same cut-off value for children and adolescents.

Using this classification, 21.3% and 14.3% of children and adolescents, respectively, had abdominal obesity in the present population. Boys were more likely to have abdominal obesity than girls, particularly in the adolescent age range. This finding is in line with data from a nationwide Greek survey of children aged 6 to 12 years [28]. Considerably lower prevalence of abdominal obesity was reported in Swedish and English children and adolescents [29], [30]. A striking contrast to these findings was reported from the NHANES survey [31]. Around one third of American children and adolescents are abdominally obese, with higher proportions among females.

There was great regional heterogeneity in the prevalence of abdominal obesity. This finding is in line with the distribution of prevalence rates for obesity in the EnKid study and the high rate of regional heterogeneity in obesity prevalence reported by the ALADINO study for children aged 6 to 9 years [32]. Different lifestyle habits between the regions of Spain might account for this finding.

Evidence indicates that the prevalence of central adiposity has increased to a higher degree than general obesity in youth [8]–[11]. Results from the Bogolusa Heart Study showed that children within normal and overweight categories with abdominal obesity were at higher cardiometabolic risk than overweight children without excessive abdominal fat [5]. This is of particular concern because classification of general obesity is the measure to identify children at high risk of obesity-related comorbidities in routine clinical practice. For this reason, we calculated the proportion of children and adolescents with abdominal obesity at low to intermediate risk according to BMI classification of normal and overweight, respectively. Additionally, the concomitant presentation of general and abdominal obesity may demand a more aggressive intervention program than would general obesity in the absence of abdominal fat accumulation. In the present study we observed a concerning proportion of children and adolescents at high risk for obesity-related comorbidities who were not classified as such by BMI criteria. Abdominal obesity based on the WHtR threshold identified more children and adolescents at high risk in normal and overweight BMI categories than did the WC threshold alone. Kromeyer-Hauschild and colleagues [33] reported a high prevalence of abdominal obesity based on WHtR in overweight adolescents aged 11 to 17 years. Abdominal obesity was more prominent in overweight boys than girls, which is in line with findings of the present study. This proportion was somewhat lower in adolescents than in children. The same was true for general obesity in the presence of abdominal obesity. In the present study, a considerable proportion of obese children and adolescents were free of abdominal obesity. The question is whether these children should be treated less “aggressively” than their abdominally obese peers.

The WHtR criteria for the characterization of abdominal obesity have generated interest because of easy calculation. Furthermore, the message of “keep your waist to half your height” is attractive for public health policy. However, a WHtR of 0.50 is not yet established as the optimal threshold for all populations and ethnicities. Indeed, two studies in different populations have reported different WHtR thresholds associated with cardiometabolic risk in children [34], [35]. More studies establishing population-specific WHtR thresholds are needed.

### Limitations and strenghts

A limitation of the present study was that pubertal status was not recored. Nonetheless, this study has several important strengths, including a nationwide population-based sample with standardized anthropometric measurements available. However, one might doubt about the actuality of the EnKid data. Recently published data from two nationally representative studies [32], [36] showed few changes in overweight and obesity prevalence rates of children and adolescents in Spain compared to the EnKid study of about a decade earlier. Although this supports the relevance of the EnKid data concerning general obesity, a disproportional increase in waist circumferences compared to BMI has been reported in pediatric populations of other countries [8]–[11]. If this change is also true for youth in Spain, then the prevalence rates reported in our study would likely be underestimated, but this remains to be tested when newer data become available.

## Conclusions

Abdominal obesity is highly prevalent in children and adolescents in Spain. Based on our results using sex- and age-specific WC cut-off values associated with high trunk fat mass, a significant proportion of normal and overweight children and adolescents were abdominally obese and can be considered at cardiometabolic risk, but would not be identified using traditional screening methods. Our results indicate the need to incorporate waist circumference into routine clinical practice, in addition to traditional measurements of weight and height.
